# Hepatoprotective Effects of *Ixora parviflora* Extract against Exhaustive Exercise-Induced Oxidative Stress in Mice

**DOI:** 10.3390/molecules180910721

**Published:** 2013-09-03

**Authors:** Nai-Wen Kan, Wen-Ching Huang, Wan-Teng Lin, Chih-Yang Huang, Kuo-Ching Wen, Hsiu-Mei Chiang, Chi-Chang Huang, Mei-Chich Hsu

**Affiliations:** 1 Graduate Institute of Athletics and Coaching Science, National Taiwan Sport University, Taoyuan 33301, Taiwan; 2 Center for Liberal Arts, Taipei Medical University, Taipei 11031, Taiwan; 3 Department of Hospitality, Tunghai University, Taichung 40704, Taiwan; 4 Graduate Institute of Basic Medical Science, China Medical University, Taichung 40402, Taiwan; 5 Department of Health and Nutrition Biotechnology, Asia University, Taichung 41354, Taiwan; 6 Department of Cosmeceutics, China Medical University, Taichung 40402, Taiwan; 7 Graduate Institute of Sports Science, National Taiwan Sport University, Taoyuan 33301, Taiwan; 8 Department of Sports Medicine, Kaohsiung Medical University, Kaohsiung 80708, Taiwan

**Keywords:** *Ixora parviflora*, oxidative stress, liver peroxidation, exhaustive exercise

## Abstract

*Ixora parviflora*, a species of the Rubiaceae, is rich in polyphenols and flavonoids, and has been traditionally used as a folk medicine. An *I. parviflora* extract (IPE) has great antioxidant activity *in vitro*, including a scavenging effect on superoxide radicals, reducing power, and ferrous ion-chelating ability. However, whether IPE is efficacious against oxidative damage *in vivo* is not known. The purpose of this study was to determine the protective effects of IPE treatment on hepatic oxidative stress and antioxidant defenses after exhaustive exercise in mice. Fifty male C57BL/6 mice (6 week old) were randomly divided into five groups and designated a sedentary control with vehicle (C), and exhaustive exercise with vehicle (IPE0), low dosage (IPE10), medium dosage (IPE50) and high dosage (IPE100) of IPE at 0, 10, 50, and 100 mg/kg, respectively. After a single bout of exhaustive swimming exercise challenge, levels of blood ammonia and creatine kinase (CK), and hepatic superoxide dismutase (SOD) protein expression, thiobarbituric acid-reactive substance (TBARS), and gp91*^phox^*, p22*^phox^*, and p47*^phox^* subunits of nicotinamide adenine dinucleotide phosphate (NADPH) oxidase expressions in the IPE0 group were significantly affected compared to those of the C group, but they were all significantly inhibited by the IPE treatments. Results of the present *in vivo* study in mice indicate that *I. parviflora* extract possesses antioxidative and hepatoprotective potential following exhaustive exercise.

## 1. Introduction

Regular and appropriate exercise benefits health, such as reducing cardiovascular disease risk, some cancers, diabetes, and osteoporosis [1]. Aerobic exercise can provide human health benefits by improving cardiorespiratory fitness which can increase the quality of life, work efficiency, musculoskeletal function, and cardiopulmonary system strength [2]. In contrast, intensive exercise can cause multiple tissue injuries due to overproduction of free radicals and reactive oxygen species (ROS). Muscular and hepatic free radical levels increase by 2–3-fold or more following exhaustive exercise [3]. Exhaustive exercise-induced liver injury is associated with unbalanced redox reactions [4], which can decrease enzymatic and non-enzymatic antioxidant levels, impair cell function and lead to eventual cell death, resulting in tissue damage and diseased states [5,6,7].

Since oxidative stress and inflammation contribute to fatigue, tissue damage, and impaired recovery from exhaustive exercise, much research has focused on supplementation with natural products to reduce those effects. *Ixora parviflora* belongs to the flavonoid-rich flowering family, the Rubiaceae, and it is mainly found distributed in China and Taiwan. Functional activities of well-known phytocompounds of *Ixora* plants, including alkaloids, saponins, steroids, alkenes, and terpenoids, were reported to include antibacterial, gastroprotective, hepatoprotective, antidiarrheal, antinociceptive, antimutagenic, antineoplastic, and chemopreventive properties [8]. In recent studies, secondary metabolites of *I. parviflora*, such as flavonoids and phenolic compounds, showed antioxidative [9], anti-inflammatory, and antiphotoaging activities [10]. However, to our best knowledge, to date there is no report on the *in vivo* bioefficacy of the antioxidative agents of *I. parviflora*. To this end, we evaluated the hepatoprotection afforded by an *I. parviflora* extract (IPE) against exhaustive exercise-induced oxidative damage in mice. We further examined the possible mechanism and provide evidence to support the antioxidant potential of the IPE in protecting the liver.

## 2. Results and Discussion

### 2.1. Effects of IPE Supplementation on Body Weight (BW), Exhaustive Swimming Time, and Fatigue-Related Biochemical Variables

BW and exercise performance data from each experimental group are summarized in Table 1. There were no significant changes in BW in any groups during the study, and no significant differences in exhaustive swimming times among the IPE0, IPE10, IPE50, and IPE100 groups. Thus, the short-term supplementation with IPE treatments did not affect body growth or improve the exercise endurance time. 

**Table 1:** Effect of *I. parviflora* extract (IPE) supplementation on body weight (BW), exercise performance, and fatigue-related biochemical characteristics after an exhaustive swimming exercise challenge.

	C	Exhaustive exercise			
		IPE0	IPE10	IPE50	IPE100
BW (g)	19.5 ± 0.6	18.2 ± 0.6	19.3 ± 1.5	18.9 ± 2.0	19.0 ± 1.2
Swimming time (s)	---	693 ± 182	749 ± 167	596 ± 185	771 ± 463
Lactate (mmol/L)	7.5 ± 3.6	9.5 ± 6.2	10.6 ± 2.8	9.2 ± 2.8	7.6 ± 2.5
NH_3_ (mmol/L)	352 ± 186 *	732 ± 249	595 ± 336	373 ± 91 *	251 ± 125 *
Glucose (mg/dL)	159 ± 55	142 ± 43	178 ± 44	207 ± 35 *	207 ± 55 *
Creatine kinase (U/L)	3349 ± 1403 *	10,940 ± 5464	8836 ± 3254	5878 ± 2294 *	5267 ± 1785 *

Mice were administrated the indicated treatments and dosages before a single bout of exhaustive exercise, and blood samples were immediately collected after exercise for biochemical analyses. Data are presented as the mean ± SD. * Indicates a significant difference with the IPE0 group (*p* < 0.05).

Biochemical parameters such as lactate, ammonia (NH_3_), glucose, and CK are important indicators of muscle fatigue after exercise [11,12,13]. After an exhaustive swimming exercise challenge, there were no significant differences in the elevation in blood lactate levels among the five groups, as illustrated in Table 1. Ammonia, a metabolite of proteins and amino acids, was linked to fatigue as early as 1922 [14]. An increase in ammonia in response to exercise can be managed by the use of glutamine and/or carbohydrates that interfere with ammonia metabolism [15]. The increase in the ammonia level is related to both peripheral and central fatigue during exercise. Respective plasma ammonia levels in the C, IPE0, IPE10, IPE50, and IPE100 groups were 352 ± 186, 732 ± 249, 595 ± 336, 373 ± 91, and 251 ± 125 µmol/L. The level of ammonia in the IPE0 group significantly increased by 2.08-fold (*p* = 0.0010) compared to the C group. It was significantly lower by 37% (*p* = 0.0018) and 58% (*p* < 0.0001) in the IPE50 and IPE100 groups, respectively, than the IPE0 group (Table 1). 

The energy supply for exercise initially comes from the breakdown of glycogen, and energy from circulating glucose is released by the liver after intense exercise [16]. Therefore, the blood glucose level is an important index for endurance performance during exercise. Levels of plasma glucose in the C, IPE0, IPE10, IPE50, and IPE100 groups were 159 ± 55, 142 ± 43, 178 ± 44, 207 ± 35, and 207 ± 55 mg/dL, respectively, and were significantly higher by 1.45- (*p* = 0.0038) and 1.46-fold (*p* = 0.0036) in the IPE50 and IPE100 groups, respectively, compared to the IPE0 group (Table 1).

The plasma level of CK can be used as an indicator to assess the degree of muscle damage [17], and previous studies showed that oxidative stress was associated with CK activity [18]. The reduction in exercise-induced oxidative damage occurs by decreasing free radical generation and blood CK activity. As shown in the Table 1, respective CK activities in the C, IPE0, IPE10, IPE50, and IPE100 groups were 3,349 ± 1,403, 10,940 ± 5,464, 8,836 ± 3,254, 5,878 ± 2,294, and 5,267 ± 1,785 U/L. The level of ammonia in the IPE0 group was significantly increased by 3.27-fold (*p* < 0.0001) compared to the C group. It was significantly lower by 46% (*p* = 0.0009) and 52% (*p* = 0.0003) in the IPE50 and IPE100 groups, respectively, compared to the IPE0 group. Therefore, IPE treatment should ameliorate skeletal muscle injury induced by acute exercise challenge.

### 2.2. Effects of IPE Supplementation on Oxidative Stress after an Exhaustive Swimming Exercise Challenge

**Figure 1:**
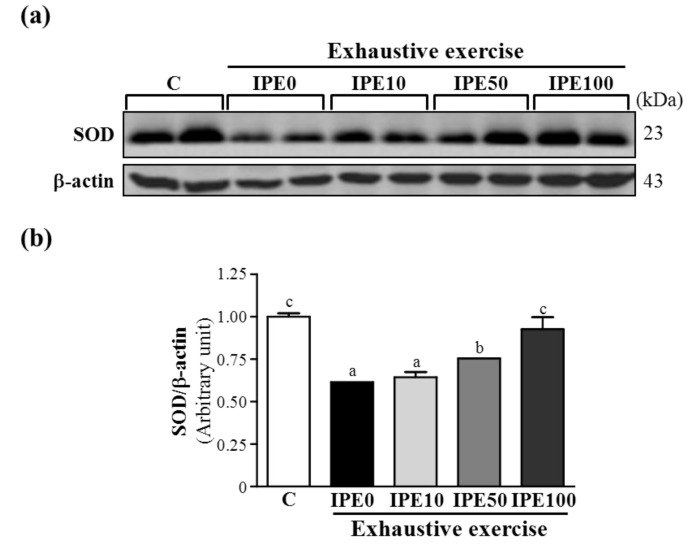
Effect of *I. parviflora* extract (IPE) supplementation on the hepatic superoxide dismutase (SOD) levels after an exhaustive swimming exercise challenge. Mice were given the indicated treatments and dosages before a single bout of exhaustive exercise, and liver samples were immediately collected after exercise for analysis of SOD expression levels. (**a**) Western blot of SOD and β-actin expression levels in the liver; (**b**) Level of SOD was normalized to β-actin.

High-intensity exercise can reduce levels of antioxidant-associated enzymes in tissues and the blood [19], enhance the production of free radicals, disturb the balance of antioxidant status, and lead to tissue injury [20]. Those studies suggested that prolonged and intense exercise increases the production of excessive free radicals and oxidative stress, which can substantially decrease antioxidant system efficiency. Therefore, appropriate antioxidant substances are likely to be vitally important in maintaining adequate antioxidant defense mechanisms [21]. Table 2 shows antioxidant enzyme activities. The various antioxidant enzyme activities such as glutathione peroxidase (GPX), glutathione reductase (GRD), and superoxide dismutase (SOD) of the liver in the IPE0 group were slightly, but not significantly lower compared to the C group. However, respective hepatic GPX, GRD and SOD activities in the IPE100 group significantly increased by 1.52-, 1.30-, and 1.30-fold, compared to that of the IPE0 group. We also examined levels of hepatic SOD by a western blot analysis. After an exhaustive swimming exercise challenge, the protein expression level of SOD in the IPE0 group was significantly lower by 38.5% (*p* < 0.0001) than that of the C group. But respective hepatic SOD expression levels in the IPE50 and IPE100 groups were significantly higher by 1.23- (*p* = 0.0083) and 1.51-fold (*p* = 0.0002) compared to the IPE0 group (Figure 1). We suggest that supplementation with the IPE prevented the exercise-induced decrease in enzymatic antioxidant defense in exhaustively exercised animals. 

**Table 2:** Effect of *I. parviflora* extract (IPE) supplementation on hepatic antioxidant enzyme activities after an exhaustive swimming exercise challenge.

Measurement	C	Exhaustive exercise			
		IPE0	IPE10	IPE50	IPE100
GPX (U/g protein)	164 ± 27	148 ± 45	162 ± 29	179 ± 54	225 ± 84 *
GRD (U/g protein)	3.3 ± 0.4	3.0 ± 0.4	3.5 ± 0.4	3.5 ± 0.7	3.9 ± 0.7 *
SOD (U/g protein)	13.7 ± 2.0	12.2 ± 2.3	14.2 ± 2.5	13.2 ± 2.4	15.8 ± 2.8 *

Mice were given the indicated treatments and dosages before a single bout of exhaustive exercise, and liver samples were immediately collected after exercise for analysis of enzyme activities. Data are presented as the mean ± SD. * Indicates a significant difference with the IPE0 group (*p* < 0.05).

A previous study demonstrated that ROS over-accumulation attacks membrane lipids after exhaustive exercise, resulting in malondialdehyde formation which affects normal cellular functions [22]. 

Data are presented as the mean ± SD. Different letters (a, b, c) indicate a significant difference at *p* < 0.05.

**Figure 2:**
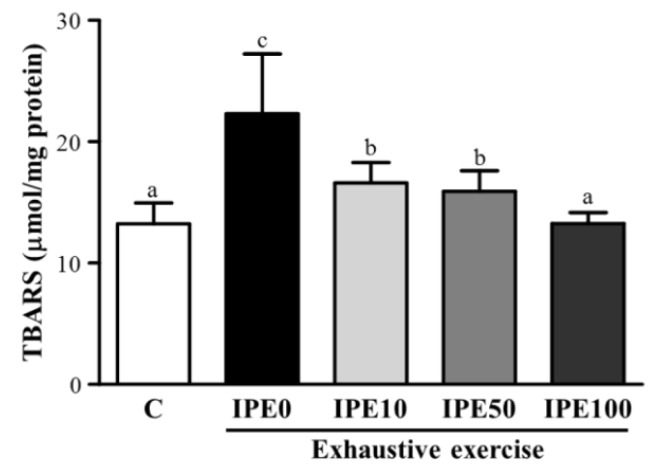
Effect of *I. parviflora* extract (IPE) supplementation on the hepatic thiobarbituric acid-reactive substance (TBARS) levels after an exhaustive swimming exercise challenge. Mice were given the indicated treatments and dosages before a single bout of exhaustive exercise, and liver samples were immediately collected after exercise for analysis of superoxide dismutase (SOD) expression.

Therefore, evaluation of lipid peroxidation in biological samples is a good marker to measure the extent of oxidative stress. The use of a thiobarbituric acid-reactive substance (TBARS) assay was carried out to measure malondialdehyde levels in blood supernatant [7] and different tissues [5]. As shown is the Figure 2, the hepatic TBARS level in the IPE0 group significantly increased by 1.68-fold (*p* < 0.0001) compared to the C group. 

Data are presented as the mean ± SD. Different letters (a, b, c) indicate a significant difference at *p* < 0.05.

But it was significantly lower by 26% (*p* < 0.0001), 29% (*p* < 0.0001), and 40% (*p* < 0.0001) in the IPE10, IPE50, and IPE100 groups, respectively, compared to the IPE0 group. These data showed that IPE supplementation effectively inhibited lipid peroxidation in the liver after an exhaustive swimming exercise challenge. 

### 2.3. Effects of IPE Supplementation on the gp91^phox^, p22^phox^, and p47^phox^ subunits of Nicotinamide Adenine Dinucleotide Phosphate (NADPH) Oxidase Complex Expression in Liver Tissue

NADPH oxidases are important sources of ROS. NADPH oxidase is comprised of many protein subunits of the enzyme complex (multicomponent enzyme), including membrane-bound proteins, such as gp22*^phox^* and gp91*^phox^*, and cytosolic proteins such as p47*^phox^*, p40*^phox^*, p67*^phox^*, and Rac1/Rac2 (GTP-binding protein). In biological systems, ROS can have both beneficial and detrimental effects. The physiological functions of ROS include host defense, cellular signaling, and cellular processes like proliferation, migration, and differentiation [23]. During NADPH oxidase activation in response to infections, cytosolic subunits are translocated to and associate with cytochrome *b*_558_, a process that results in oxidase activation to initiate the defense system [24]. However, increased ROS levels due to NADPH oxidase activation also contribute to a wide range of pathological processes and lead to tissue injuries. In this study, we also investigated whether NADPH oxidase protein expressions were associated with the liver oxidative stress level response to an exhaustive exercise challenge. 

**Figure 3:**
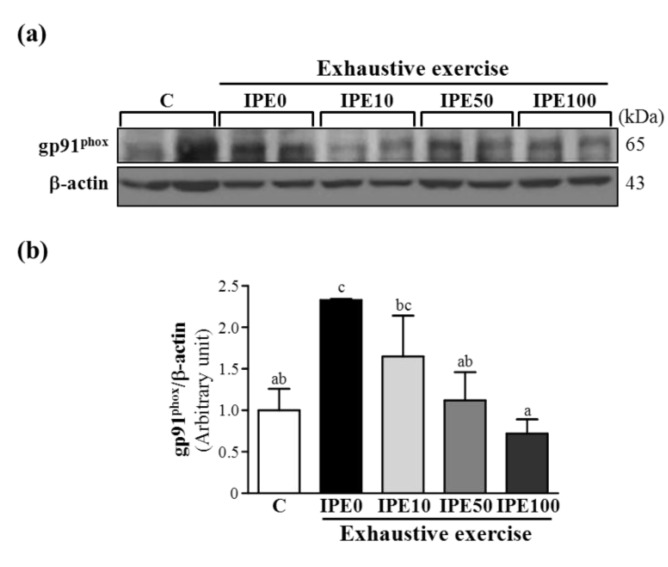
Effect of *I. parviflora* extract (IPE) supplementation on levels of the gp91*^phox^* subunit of NADPH oxidase in liver tissues after an exhaustive swimming exercise challenge. (**a**) Western blot of gp91*^phox^* and β-actin expression levels in the liver; (**b**) gp91*^phox^* protein expression was normalized to that of β-actin (internal control) by densitometry.

Data are presented as the mean ± SD. Different letters (a, b, c) indicate a significant difference at *p* < 0.05.

**Figure 4:**
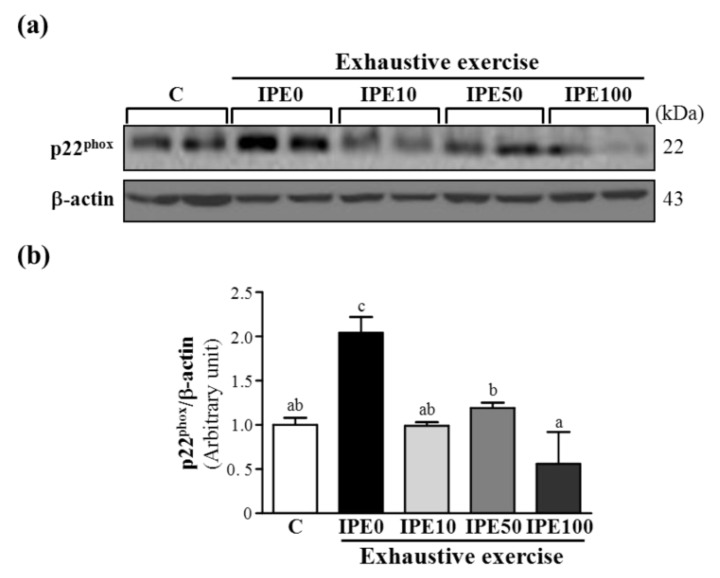
Effect of *I. parviflora* extract (IPE) supplementation on levels of the p22*^phox^* subunit of NADPH oxidase in liver tissues after an exhaustive swimming exercise challenge. (**a**) Western blot of p22*^phox^* and β-actin expression levels in the liver; (**b**) p22*^phox^* protein expression was normalized to that of β-actin (internal control) by densitometry. Data are presented as the mean ± SD. Different letters (a, b, c) indicate a significant difference at *p* < 0.05.

**Figure 5:**
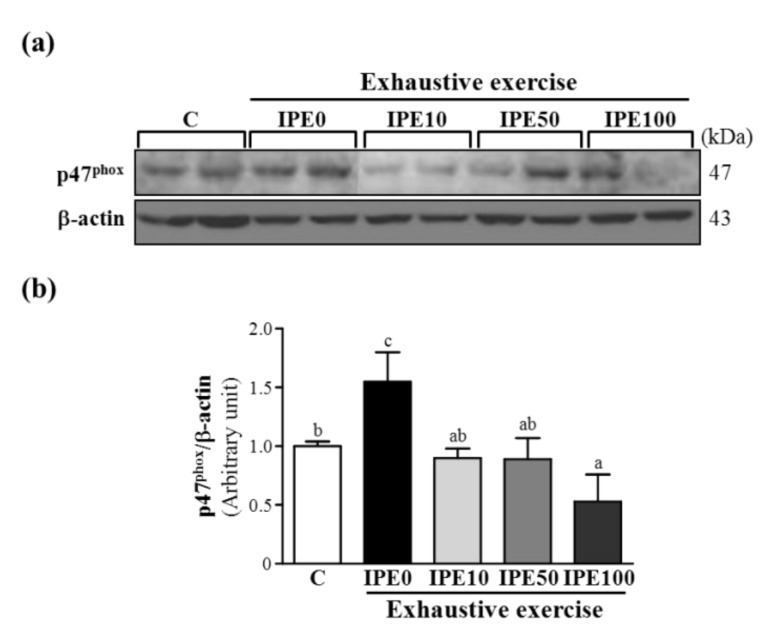
Effect of *I. parviflora* extract (IPE) supplementation on levels of the p47*^phox^* subunit of NADPH oxidase in liver tissues after an exhaustive swimming exercise challenge. (**a**) Western blot of p47*^phox^* and β-actin expression levels in the liver; (**b**) p47*^phox^* protein expression was normalized to that of β-actin (internal control) by densitometry.

As shown in Figure 3, Figure 4 and Figure 5, after an exhaustive swimming exercise challenge, protein expression levels of hepatic gp91*^phox^*, p22*^phox^*, and p47*^phox^* subunits in the IPE0 group significantly increased by 2.33- (*p* = 0.0067), 2.04- (*p* = 0.0025), and 1.55-fold (*p* = 0.0267), respectively, compared to those of the C group. Compared to the IPE0 group, hepatic gp91*^phox^*, p22*^phox^*, and p47*^phox^* expression levels in the IPE10 group slightly and significantly decreased by 29.15% (*p* = 0.0723), 51.51% (*p* = 0.0024), and 42.00% (*p* = 0.0145), respectively. Moreover, the hepatic gp91*^phox^*, p22*^phox^*, and p47*^phox^* expression levels in both the IPE50 and IPE100 groups all significantly decreased compared to those of the IPE0 group. Our observations suggest that IPE supplementation effectively inhibited hepatic NADPH oxidase expression, which could contribute to decreasing oxidative stress after an exhaustive swimming exercise challenge. 

Data are presented as the mean ± SD. Different letters (a, b, c) indicate a significant difference at *p* < 0.05.

### 2.4. Proposed Mechanisms by Which IPE Acts against Oxidative Stress Due to Exhaustive Exercise

**Figure 6:**
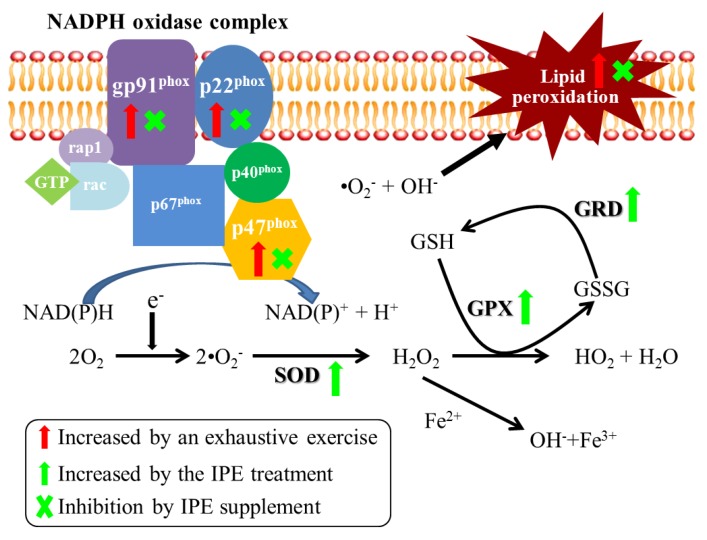
Summary of proposed mechanisms of *I. parviflora* extract (IPE) supplementation that served to protect mice from a single bout of exhaustive exercise-induced oxidative stress. The red (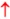) and green arrows (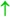) represent upregulation by exhaustive exercise challenge and IPE treatment, respectively. The (**×**) symbol represents inhibition by IPE treatment.

In the present study, we observed a reduction in antioxidant enzyme expressions, and increases in lipid peroxidation and expressions of the gp91*^phox^*, p22*^phox^*, and p47*^phox^* subunits of NADPH oxidase in animals after exhaustive exercise, while IPE supplementation blocked the lipid peroxidation and upregulation of NADPH oxidase expression. We therefore propose herein that the IPE can protect against exhaustive exercise-induced oxidative stress through deregulation of the NADPH oxidase expression and upregulation of antioxidative enzyme activity and expression (Figure 6).

## 3. Experimental

### 3.1. Plant Material and Extraction

*Ixora parviflora* leaves were cleaned by washing twice with deionized distilled water and dried in an oven at 50 °C. The dried leaves were finely pulverized, and 1,015 g was weighed for extraction with methanol (30 L) with 60 min of infiltration. Samples were further processed by 1 h of sonication, and filtered through a Buchner funnel. These steps were repeated twice, and the methanol solvent was combined twice for rotoevaporation. The obtained residue, defined as the IPE, was dried under vacuum and stored in an electronic dry cabinet for the following experiments. The detailed chemical and physical characteristics of IPE have been reported in the previous study [9].

### 3.2. Animals, Treatment, and Exhaustive Swimming Exercise Challenge

Fifty male C57BL/6 mice (6 weeks old) were purchased from an animal breeding and research center (National Taiwan University, Taipei, Taiwan). All animals were given a standard laboratory diet (no. 5001; PMI Nutrition International, Brentwood, MO, USA) and distilled water *ad libitum* and appropriately housed in a room maintained at 24 ± 1 °C and humidity of 55% ± 10% with a 12 h light-dark cycle. Before the experiments, mice were raised for 1 week to allow acclimation to the environment and diet. The Institutional Animal Care and Use Committee (IACUC) of National Taiwan Sport University (NTSU) inspected all animal experiments in this study, and this study conformed to guidelines of protocol IACUC-10003, approved by the IACUC ethics committee.

All animals were randomly divided into five groups (ten mice per group) for IPE treatment as follows: (1) sedentary control with vehicle (C); (2) 0 mg/kg IPE (IPE0); (3) 10 mg/kg IPE (IPE10); (4) 50 mg/kg IPE (IPE50); and (5) 100 mg/kg IPE (IPE100). Test animals were pretreated intraperitoneally (*i.p.*) with each treatment for three consecutive days and 1 h before a single bout of exhaustive exercise to evaluate the protective effect of IPE. Mice in the IPE0, IPE10, IPE50, and IPE100 groups performed an exhaustive swimming exercise challenge as previously described [13]. Before the exhaustive exercise experiment, mice were starved for 8 h. A weight equivalent (5% of body weight) was attached to the mouse tail, and the endurance of each mouse in the different treatments was measured as swimming times recorded from the beginning to exhaustion. When a mouse was unable to reach the water surface for 7 s, it was defined as being exhausted. Blood samples were collected immediately after the exhaustive exercise, and then all mice were killed and liver tissues were collected. 

### 3.3. Determination of Blood Biochemical Variables

To evaluate the potential anti-fatigue effects of IPE treatment, the plasma lactate, ammonia, glucose, and CK levels were analyzed as in our previous study [11]. Blood samples were collected from mice immediately after the exhaustive swimming exercise. Plasma was prepared by centrifugation at 1500 ×*g* for 10 min at 4 °C, and then analyzed with a Hitachi 7060 autoanalyzer (Hitachi, Tokyo, Japan).

### 3.4. Analysis of Oxidative Stress-Associated Parameters in Liver Tissues

Activities of the antioxidant enzymes, glutathione peroxidase (GPX), glutathione reductase (GRD) and superoxide dismutase (SOD) and thiobarbituric acid-reactive substance (TBARS) levels, an index of lipid peroxidation and oxidative stress, in liver tissues were analyzed according to our recent study [7]. Total protein concentrations of samples were determined using a DC protein assay kit (Bio-Rad Laboratories, Hercules, CA, USA). The specific activity of test enzymes was expressed as U/g protein. 

### 3.5.Immunoblotting

Primary antibodies against antioxidation-associated proteins including SOD (Santa Cruz Biotechnology, Santa Cruz, CA, USA), p22^phox^ (BD Biosciences, Piscataway, NJ, USA), gp91^phox^ (Santa Cruz), and p47^phox^ (BD Biosciences), were used for the western blot analysis. All other antibodies including second antibodies and internal control (β-actin) were from Santa Cruz. The western blot analysis followed a previous report [25]. The protein content was measured by the Bradford method (Bio-Rad). Proteins were resolved by 5%–20% gradient sodium dodecylsulfate (SDS)-polyacrylamide gel electrophoresis (PAGE) and then immunoblotted using an enhanced chemiluminescence (ECL) assay (Perkin Elmer Life Science, Boston, MA, USA) with image retrieval using the Gel analysis system (EverGene Biotechnology, Taipei, Taiwan). The intensities were quantified using Phoretix 1D software (Phoretix International, Newcastle upon Tyne, UK).

### 3.6. Statistical Analysis

All data are represented as the mean ± SD. To evaluate differences among the groups studied, data were analyzed using a one-way analysis of variance (ANOVA) with the Statistical Analysis System vers. 9.0 (SAS Institute, Cary, NC, USA). *p* < 0.05 was considered statistically significant.

## 4. Conclusions

Our data demonstrated that the *I. parviflora* extract increased the antioxidant enzyme activities and SOD protein expression, decreased lipid peroxidation, and inhibited the NADPH oxidase complex expression in the liver after exhaustive exercise challenge. These results indicate that the *I. parviflora* extract has antioxidative and liver-protective effects *in vivo*. Although the exact bioactive phytocompounds in *I. parviflora* that contribute to the detailed antioxidative mechanisms and hepatoprotection remain to be elucidated, this study provides science-based evidence to support the fact that *I. parviflora* is possibly a promising antioxidant affording liver protective effects during exercise.
